# Marked Improvement in Severe Chronic Penile Pain Following the Administration of Mirogabalin: A Case Report

**DOI:** 10.7759/cureus.65231

**Published:** 2024-07-23

**Authors:** Shinju Obara, Yuko Nakano, Reiko Oishi, Satoki Inoue

**Affiliations:** 1 Anesthesiology, Fukushima Medical University, Fukushima, JPN

**Keywords:** balanitis, treatment choices, pain clinic, mirogabalin, chronic penile pain

## Abstract

Chronic penile pain (CPP) is a debilitating condition characterized by persistent penile pain, often accompanied by lower urinary tract symptoms and sexual dysfunction, severely impacting quality of life. Treatment options are limited. We report a case of a 38-year-old man with severe CPP effectively treated with mirogabalin. Initially diagnosed with balanitis, his symptoms evolved to include severe penile pain and mental instability, and he was unresponsive to standard treatments. After multiple failed therapies, mirogabalin was introduced, resulting in significant pain reduction and improved mental health within days. This case highlights mirogabalin's potential efficacy in treating CPP with neuropathic characteristics, suggesting it as a viable treatment option for similar cases. Further studies are warranted to explore its broader applicability.

## Introduction

Chronic penile pain (CPP) is defined as persistent or recurrent pain in the penis that is not primarily located in the inside of the urethra and may occur spontaneously or be reproduced by physical activity. It can also cause lower urinary tract symptoms and sexual dysfunction [1], leading to a significant decline in quality of life. However, treatment options for penile pain are limited [2]. Herein, we describe a case of severe CPP that was effectively treated using mirogabalin.

## Case presentation

A 38-year-old man with a history of taking privately imported dutasteride to prevent hair loss was diagnosed with balanitis with itching on the glans at his local clinic and was under treatment with steroid ointments. Sixteen months after diagnosis of balanitis, he noticed cloudy urine. He was diagnosed as having prostatitis at a different clinic and was treated with levofloxacin for three months. Subsequently, the irritation in the glans and frenulum preputii became progressively more severe. He began experiencing insomnia and mental instability, and a somatoform disorder was suspected, but he refused psychiatric treatment. He even requested partial penile resection. He underwent a urological workup that included tests for sexually transmitted infections, but all were negative. Oral hydroxyzine and acetaminophen were ineffective. The patient quit his job and was referred to our pain clinic 45 months after diagnosis of balanitis.

The patient’s only visible symptom was mild redness in a small area at the tip of the glans, but his pain extended to the glans, frenulum preputii, and mid-penis. His pain intensity was 1/10 on the Numerical Rating Scale for pain, but he stated that at night the score increased to 10/10 and was accompanied by itching. His scores on the Pain Catastrophizing Scale [3] and the Hospital Anxiety and Depression Scale [4], both in the Japanese version, were 47 and 19 (Anxiety 8 and Depression 11), respectively, indicating catastrophic thinking and depression. He described the nature of the pain as intermittent, intense, electric, and stabbing, which was suggestive of neuropathic pain. Mirogabalin 10 mg/day was administered, resulting in gradual improvement of his pain over a period of seven days, leaving only mild itching. Mirogabalin was increased to 20 mg/day, and the itching further improved in a few days. The patient has since been followed up with the same dose of mirogabalin for three months without adverse events. His mental status and insomnia have dramatically improved, and he has begun to look for a new job.

## Discussion

CPP is sometimes treated symptomatically with analgesics when no cause, such as organic abnormalities or evidence of infection, is found. However, if analgesics are ineffective, psychiatric treatment may become the preferred option. It is noteworthy that the rate of depression in this patient population is nearly 20% higher than the average [2]. To the best of our knowledge, this is the first report showing the significant efficacy of treatment with mirogabalin in a case of CPP with itching. Similarly, there are no such reports for pregabalin, which is another de facto standard for neuropathic pain.

Sensory innervation of the penis and glans is supplied by the dorsal nerve (DN), which is a terminal branch of the pudendal nerve [1]. In the present case, chronic inflammation from prolonged glomerulitis, typically associated with nociceptive pain, may have damaged free nerve endings of the DN, leading to symptoms indicative of neuropathic pain. Additionally, neuropathic itch is known to occur when small nerve fibers sustain damage. Mirogabalin, by inhibiting calcium influx in the peripheral nervous system and suppressing neurotransmitter release, exerts analgesic effects [5]. Its potential efficacy in alleviating itching has also been recognized [6]. Although pregabalin has been reported to be effective for neuropathic itching [7], we opted for mirogabalin based on the attending anesthesiologists' discretion. The patient stated that as a result, his insomnia was improved due to the side effect of somnolence [8].

Furthermore, it is important to note that chronic prostatitis can cause persistent penile and perineal pain. However, its direct connection to the CPP observed in this case remains uncertain.

Watson and Zhu reported a case of CPP that was successfully managed with a DN block [2]. Additionally, more invasive treatment options have been proposed, including pulsed or thermal radiofrequency ablation and cryoablation of the DN [9]. If oral medications had been ineffective in the present case, we may have considered these options.

## Conclusions

This case report demonstrates that mirogabalin effectively treated severe CPP, leading to significant improvements in pain, itching, and associated symptoms such as insomnia. The patient's quality of life markedly improved, allowing him to resume daily activities and search for a job. These results suggest that mirogabalin may be a promising treatment option for CPP, particularly when other analgesics are ineffective.
